# Determinants of cesarean mode of childbirth among Rwandan women of childbearing age: Evidence from the 2019–2020 Rwanda Demographic and Health Survey (RDHS)

**DOI:** 10.1002/puh2.150

**Published:** 2024-01-30

**Authors:** Nsereko Etienne, Uwase Aline, Mpinganzima Ornella, Usanzineza Henriette, Niyitegeka Jean Pierre, Turabayo Jean Léonard, Mwiseneza Marie Josee, Mugeni Girimpundu Candide, Moreland Patricia

**Affiliations:** ^1^ School of Health Sciences, University of Rwanda College of Medicine and Health Sciences Kigali Rwanda; ^2^ School of Nursing and Midwifery, University of Rwanda College of Medicine and Health Sciences Kigali Rwanda; ^3^ Lillian Carter Center for Global Health and Social Responsibility, Nell Hodgson Woodruff School of Nursing, Emory University Atlanta Georgia USA

**Keywords:** cesarean delivery, clinical factors, pregnancy, private–public health facilities, socioeconomic status

## Abstract

**Background:**

Rwanda has made progress in reducing maternal mortality, with rates decreasing by three‐quarters between 1990 and 2015, in part due to increased access to cesarean delivery (CD). However, the prevalence of CD is also increasing for reasons other than clinical indications. Rwanda's 15% CD delivery rate is higher than World Health Organization guidelines, sparking debate about optimal CD use and potential negative outcomes in child and maternal health. This study aimed to identify the key clinical and nonclinical factors relating to CD in Rwanda.

**Methods:**

A secondary data analysis of the Rwanda Demographic and Health Survey (2019–2020) was performed, using the outcome variable of vaginal birth versus CD in women who had delivered in the survey period and for 5 years preceding. We used logistic regression modeling to identify the explanatory sociodemographic, economic, and obstetric characteristics associated with CD.

**Results:**

More than half of participants were between 20‐ and 34‐year old, 65.2% had completed primary school at least, 79.0% lived in rural areas, and 81.2% had health insurance. Fifteen percent of participants had given birth by CD in the preceding 5 years. CD was independently associated with higher levels of education, religion, higher socioeconomic status, perceived financial constraints, and giving birth in a private health facility. Obstetric predictors for CD included twin pregnancy, male infancy, and primiparity.

**Conclusion:**

Results suggest that indications for CD should be monitored and the birthing preferences of women further evaluated, with guidelines implemented to inform decision‐making around CD.

## Graphical abstract

We found that cesarean birth in Rwanda was associated with sociodemographic predictors of higher education level, higher socioeconomic status, religious beliefs, and access to private health facilities; obstetric predictors included primary cesarean section, higher baby size, and twins.

The birthing preferences of women in Rwanda need further evaluation; indications for cesarean section need to be monitored and policy guidelines developed.

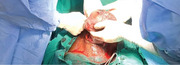

## INTRODUCTION

Maternal and infant mortality remain high in developing countries, particularly in sub‐Saharan Africa, which accounts for two thirds of all maternal deaths worldwide [1, 2]. Rwanda has made remarkable progress in reducing maternal mortality, falling by three‐quarters between 1990 and 2015. This success has been the result of a combination of policies and interventions, including increased access to cesarean delivery (CD), with the proportion of deliveries by CD in Rwanda increasing from 2% in 1992 to 15% in 2019–2020 [3, 4, 5].

CD is a life‐saving obstetric surgery for both mother and child, and the World Health Organization (WHO) recommends that CD should be available when clinically indicated [6]. The WHO does not set a population‐level target rate, but data indicate that CD rates above 10% do not further reduce mother and neonatal mortality [7].

Previous research has found that institutional births in Rwanda, particularly in private facilities, are increasingly likely to be by CD, and the Rwanda Demographic and Health Survey (RDHS) has demonstrated a similar trend over the last three decades [8]. Furthermore, it was discovered that educated women living in urban areas and of high socioeconomic status (SES) are more likely to deliver by CD than their counterparts with low education levels, living in rural areas, and of low SES [9, 10, 11]. CD rates are increasing for a variety of reasons aside from clinical need. Maternal preference and fear of complications associated with vaginal delivery are among the reasons cited [12, 13]. Evidence suggests that nonclinical factors may also include financial gain for healthcare institutions, as CD is more lucrative than vaginal delivery [14, 15]. There is a growing perception that CD is both underutilized and overutilized within and across countries and continents [16, 17], which has sparked a heated debate among professionals worldwide about optimal CD delivery rates [18, 19, 20, 21]. Data show that the prevalence of CD varies by country, accounting for 43% of births in South America and 5% in Africa [22]. In Rwanda, the private sector has a CD rate of 28.1% compared to 12% in the public sector, with an overall rate of 15% [3].

Several studies have found that high CD rates are associated with negative outcomes for both mother and child. CD has been linked to long‐term maternal morbidity, although most studies have focused on short‐term rather than long‐term complications [16, 19, 23]. As a result of difficulties in obtaining long‐term follow‐up data, the literature is mostly focused on chronic complications, such as surgical adhesions, infertility, and subfertility, as well as the life‐threatening risks of hemorrhage and hysterectomy [24, 25]. It has also been reported that the risk of perinatal complications and other long‐term morbidities increases with the number of cesarean procedures performed, with the risk being highest in women who have had multiple cesareans [26, 27].

The 15% rate of CD in Rwanda is higher than WHO guidelines. Factors associated with CD in Rwanda are only partially documented, and hospital‐based studies have been insufficient to support the development of evidence‐based policies. This study aimed to identify key factors from a community‐based database, in preparation for extensive hospital‐based research on clinical and nonclinical CD indications in Rwanda.

## METHODS

This study was a secondary data analysis of the most recent RDHS (2019–2020), a countrywide cross‐sectional survey conducted every 5 years by the Rwandan government with support from international partners. Using a two‐stage sample design, the RDHS estimates key health indicators at the national, urban, and rural levels, as well as for five provinces and each of the 30 districts in Rwanda. First, sample sites (clusters) of delimited enumeration areas (EAs) were selected. The second stage involved systematic household sampling. The household lists of the selected EAs were used to choose survey participants at random. The RDHS collects data using various questionnaires; the data of interest in this study were collected using the Woman Questionnaire, where women aged from 15 to 49 years were the primary respondents, answering detailed questions about themselves, their households, and their children.

### Outcome variables

In the RDHS, women who had delivered in the 5 years preceding the survey were asked if they had delivered through a cut of the belly to take the baby out. The answer ‘Yes’ indicated CD delivery, whereas the answer ‘No’ indicated vaginal delivery. Thus, the outcome variables in this dataset clearly indicated vaginal versus CD.

### Explanatory variables

A wide range of explanatory variables were derived from the literature, including sociodemographic and obstetric characteristics. For sociodemographic data, maternal age at the time of birth was divided into three categories: less than or equal to 19 years, 20–34 years, and 35 years old and above. The region of residence included the five provinces in Rwanda (Kigali, South, West, North, and East), and the type of residence was categorized as urban or rural. Religion was categorized into Catholic, Protestant, Adventist, Muslim, and others. Women's education was divided into four levels: no education, primary, secondary, and higher education. Participants were classified as employed or unemployed based on their employment status. Marital status was classified as never in union, married or staying with a partner, widowed or divorced, or separated. The wealth category was divided into poorest, poorer, middle, richer, and richest. Health insurance coverage was divided into two categories: covered and not covered. Perceived distance to a healthcare facility was categorized into “not a problem” versus “a problem.” Perceived difficulties in paying for treatment were categorized into “not a problem” versus “a problem.” Place of delivery was divided into public versus private health facilities. Maternal Body Mass Index (BMI) was categorized into underweight, normal weight, and overweight/obese. In terms of obstetric characteristics, the following variables were considered: total number of children ever born (categorized as one child, two to four children, and five or more children). Birth order was classified as first child, second or third child, and fourth or higher child. Birth status (single vs. multiple birth) was divided into single birth and twin birth. Size at birth was classified as very large, larger than average, average, small, and very small. The gender of the baby was coded as female (1) or male (0).

### Data analysis

SPSS version 20 was used to analyze the data, and descriptive statistical results were shown in frequency tables. The chi‐squared test was used in univariate analysis to identify potential explanatory variables associated with CD. To account for sample variation, we used design‐based weighting in accordance with RDHS guidelines (V005/1000000). With regards to independent sociodemographic, economic, and obstetric characteristics associated with CD, multiple logistic regression models were created, including any variable with a *p*‐value of at least 0.05 (two‐sided). Following that, a backward‐stepwise regression model was used to manually decide whether to keep or remove a variable from the model based on likelihood ratio test and the chi‐squared value in the Hosmer and Lemeshow goodness‐of‐fit test, where *p* > 0.05 and a smaller chi‐squared value indicated a good model fit. The study controlled for demographic characteristics in each series of regression models to control for a clustering effect of demographic and household characteristics.

In the final phase, an automatic backward‐stepwise regression analysis was performed, yielding an adjusted model with a *p*‐value of at least 0.05 (two‐sided) set as the level of significance. Findings are presented in the form of odds ratios (ORs) with 95% confidence intervals (CIs).

### Ethical considerations

This study was a secondary data analysis from the RDHS, a large‐scale national survey approved by the Macro International, the Rwandan National Institute of Statistics, and the National Ethics Review Board in Rwanda. In addition, to access the dataset, consent was obtained via an online application. The dataset was only used for analysis, and contact with the study participants was not required.

## RESULTS

There were 8082 responses regarding the mode of delivery (CD vs. vaginal delivery), with 1294 (15%) giving birth via CD. The majority of participants (63.2%, *n* = 5111) were between 20‐ and 34‐year old, had completed only primary school education (65.2%, *n* = 5401), had normal weight (67.4%, *n* = 2750), had no problems paying for treatment (54.3%, *n* = 4393), had two to four children (57.2%, *n* = 4626), and gave birth to average‐sized babies (52.1%, *n* = 4188). Most participants (79.0%, *n* = 6390) were from rural areas, employed (75.2%, *n* = 6083), and did not perceive the distance to the healthcare facility as a key challenge (76.4%, *n* = 6183). The majority of the participants were married/staying with a partner (83.2%, *n* = 6734), had health insurance (81.2%, *n* = 6569), had singleton deliveries (98.5%, *n* = 7851), and delivered in public health facilities (97.7%, *n* = 7361). Fewer than half (44.2%, *n* = 3576) belonged to the two lowest wealth quartiles (poorer and poorest), were Protestant (49.3%, *n* = 3986), and delivered female children (49.4%, *n* = 3997). In the univariate analysis, CD was associated with all sociodemographic and obstetric factors considered in this study (Table 1).

**TABLE 1:** Social demographics and obstetrics characteristics of the participants.

	Total (*n* = 8092)		
Variables	*N*	%†	*χ* ^2^ *p*‐Value
**Mode of delivery**		
Vaginal	6858	84.8	
Cesarean delivery (CD)	1234	15.2	
**Age at the time of giving birth (*n* = 8092)**			0.03
≤19 years	125	1.5	
20–34 years	5111	63.2	
≥35 years	2856	35.3	
**Region (*n* = 8092)**			0.01
Kigali	948	11.7	
South	1853	22.9	
West	2069	25.6	
North	1282	15.8	
East	1940	24.0	
**Religion (*n*: 8092)**			0.01
Catholic	2775	34.3	
Protestant	3986	49.3	
Adventists	1031	12.7	
Muslim	155	1.9	
Others	145	1.8	
**Residence type (*n* = 8092)**			<0.001
Urban	1702	21.0	
Rural	6390	79.0	
**Women education (*n* = 8092)**			<0.001
No education	918	11.3	
Primary	5277	65.3	
Secondary	1507	18.6	
Higher	390	4.8	
**Employment status (*n* = 8092)**			<0.001
Not employed	2009	24.8	
Employed	6083	75.2	
**Marital status (*n* = 8092)**			
Never in union	719	8.9	
Married/Staying with a partner	6734	83.2	
Widower/Divorced/Separated	639	7.9	
**Wealth category (*n*: 8092)**			<0.001
Poorest	2007	24.8	
Poorer	1569	19.4	
Middle	1518	18.8	
Richer	1510	18.6	
Richest	1488	18.4	
**Health insurance coverage** (***n* = 8092**)			
No	1523	18.8	
Yes	6569	81.2	
**Maternal BMI (*n* = 4081)**			0.01
Underweight	134	3.3	
Normal weight	2750	67.4	
Overweight and obese	1197	29.3	
**Perceived distance to health facility (*n* = 8092)**			
Not a problem	6183	76.4	
Big problem	1909	23.6	
**Perceived difficulties in getting money for treatment (*n* = 8092)**			<0.001
Not a problem	4393	54.3	
Big problem	3699	45.7	
Total children ever born (parity) (*n* = 8092)*			<0.001
First born	1478	18.3	
Two to four children	4626	57.2	
Five or more	1988	24.5	
Birth status (twins) (*n*: 7972)*			<0.001
Not twin	7851	98.5	
Twin	121	1.5	
**Size at birth (*n* = 8045)**			0.03
Very large	444	5.5	
Larger than average	1949	24.2	
Average	4188	52.1	
Small	1464	18.2	
**Place of delivery (*n* = 7532)**			<0.001
Public HF	7361	97.7	
Private HF	171	2.3	
**Baby sex (*n*: 8092)**			0.03
Male	4095	50.6	
Female	3997	49.4	

*stand for missing values.

The following independent predictors of CD were identified by the multivariable logistic regression: starting with religion, compared to Catholics, being Protestant (OR = 1.22, CI = 1.06–1.41, *p* = 0.05), or Muslim (OR = 1.64, CI = 1.10–2.45, *p* = 0.01) was significantly associated with CD.

With regards to education, participants who completed primary education (OR = 0.42, CI = 0.29–0.60, *p* < 0.001), secondary education (OR = 0.43, CI = 0.37–0.63, *p* < 0.001), or higher education (OR = 0.48, CI = 0.37–0.63, *p* < 0.001) were less likely to have undergone CD compared to their counterparts with no education.

With regards to wealth, participants in the wealthier quintiles, specifically richer (OR = 1.36, CI = 1.10–1.68, *p* < 0.001) and richest (OR = 2.03, CI = 1.61–2.55, *p* < 0.001), had a higher likelihood of CD compared to their counterparts from the lower quintiles.

Similarly, having health insurance coverage increased the likelihood of CD being performed (OR = 1.21, CI = 1.00–1.46, p = 0.049) as well as the participant's perception of difficulties in paying for healthcare services (OR = 1.15, CI = 1.00–1.46, *p* = 0.04).

Other independent factors for increased likelihood of CD were the number of children ever born, including having five or more children (OR = 0.52, CI = 0.43–0.67, *p* < 0.001), twin status (OR = 3.16, CI = 2.06–4.86, *p* < 0.001), delivering in private healthcare facilities (OR = 2.58, CI = 1.83–3.65, *p* < 0.001), and the female sex of the child (OR = 0.87, CI = 0.77–0.99, *p* = 0.035; Table 2).

**TABLE 2:** Multiple logistic regression analysis models.

	Full model			Adjusted model		
Variables	Odds ratio (OR)	95% CI	*p*‐Value	Odds ratio (OR)	95% CI	*p*‐Value
**Age at the time of giving birth**						
≤19 years	1					
20–35 years	0.81	0.42–1.55	0.52			
>35 years	0.95	0.47–1.89	0.87			
**Region (*n* = 8092)**						
Kigali	1					
South	1.25	0.90–1.74	0.17			
West	0.94	0.68–1.29	0.70			
North	0.73	0.68–1.29	0.10			
East	0.93	0.66–1.29	0.65			
**Religion**						
Catholic	1			1		
Protestants	1.09	0.89–1.33	0.39	1.22	1.06–1.41	0.005
Adventists	0.99	0.73–1.34	0.95	1.09	0.88–1.35	0.40
Muslim	1.96	1.17–3.29	0.01	1.64	1.10–2.45	0.015
Others	0.51	023–1.12	0.09	0.75	0.43–1.34	0.34
**Residence**						
Urban	1					
Rural	0.93	0.72–1.19	0.56			
**Women education**						
No education	1			1		
Primary	0.54	0.32–0.89	0.018	0.42	0.29–0.60	<0.001
Secondary	0.53	0.35–0.79	0.002	0.43	0.33–0.56	<0.001
Higher	0.71	0.48–1.059	0.094	0.48	0.37–0.63	<0.001
**Employment status**						
Not employed						
Employed	0.95	0.77–1.17	0.65			
**Marta status**						
Never in union	1					
Married/Staying with a partner	0.98	0.71–1.35	0.90			
Divorced/Widowed/Separated	0.93	0.60–1.46	0.76			
**Wealth category**						
Poorest	1			1	1	
Poorer	0.96	0.71–1.29	0.79	0.93	0.75–1.16	0.54
Middle	1.08	0.79–1.47	0.61	1.065	0.85–1.32	0.57
Richer	1.11	0.80–1.50	0.56	1.36	1.10–1.68	0.004
Richest	1.52	1.06–2.19	0.023	2.03	1.61–2.55	<0.001
**Health insurance coverage**						
No				1		
Yes	1.37	1.04–1.81	0.024	1.21	1.00–1.46	0.049
**Maternal BMI**						
Underweight	1					
Normal weight	0.74	0.44–1.22	0.23			
Overweight/Obese	1.10	0.62–1.74	0.89			
**Perceived distance to health facilities**						
Not a big problem	1					
A big problem	0.81	0.63–1.04	0.09			
**Perceived difficulties in getting money for treatment**						
**Not a big problem**	1			1		
A big problem	1.15	0.93–1.42	0.20	1.15	1.00–1.46	0.04
**Total children ever born**						
First born	1			1		
Two to four children	0.89	0.69–1.14	0.38	0.93	0.79–1.09	0.37
Five or more	0.55	0.38–0.79	0.002	0.52	0.43–0.67	<0.001
**Birth status**						
Not twins	1			1		
Twins	3.27	1.79–5.97	<0.001	3.16	2.06–4.86	<0.001
**Size at birth**						
Very large	1					
Larger than average	0.81	0.55–1.18	0.27			
Average	0.69	0.48–0.98	0.04			
Small	0.70	0.47–1.06	0.09			
**Place of delivery**						
Public HF	1			1		
Private HF	2.57	1.53–4.31	<0.001	2.58	1.83–3.65	<0.001
**Sex of the child**						
Male	1			1		
Female	0.82	0.68–0.97	0.028	0.87	0.77–0.99	0.035

## DISCUSSION

Our secondary data analysis of 8092 women from a nationally representative sample revealed a CD prevalence rate of 15%, indicating a decade‐long increase in CD prevalence in Rwanda. Increased CD prevalence is a worldwide trend that has been observed in a number of other countries [9, 15, 28, 29]. In our study, the risk of CD was decreasing with maternal education but increases with higher wealth status and in women giving birth in private healthcare facilities. Similar findings have been made in previous studies [9, 15, 20, 30]. Maternal age was not associated with CD, which contrasts with findings from other publications [31, 32, 33]. The discrepancy might result from using different age categories; in our case, we used the WHO definition of high and low‐risk pregnancies [34]. The finding of an association between religion and CD is not easy to understand. However, other studies have documented that religious ideology impacts social norms, beliefs, and expectations [35]. Therefore, in this study, it is possible that religious factors influenced the birth choices of participants for various reasons, including delays in health‐seeking behavior resulting from gender‐based imbalances observed in some regions.

This study found an association between level of education and likelihood of CD, with higher education levels conferring reduced risk. These findings are in line with other studies showing that a high level of maternal education is associated with a lower risk of CD [9, 15]. However, the reason for this association is unknown. One study found that maternal health service utilization was related to educational level [36]. This suggests that a high level of education improves access to healthcare services, where good pregnancy surveillance prevents complications that lead to CD, in particular emergencies. According to the present study findings, women from wealthy families were more likely to have CD than those from low‐income families, and this is consistent with previous research [10, 12, 15, 37]. Various studies have suggested that financial constraints in the developing world contribute to the unavailability of many healthcare interventions, including CD [38]. Given that CD is one of the most expensive healthcare interventions in Rwanda, it is possible that women from wealthier families have better financial access than their counterparts from resource‐limited families in this study context [39]. In the same way, this study found a link between emergency cesarean delivery and women saying they had trouble paying for medical care. This shows how hard it is for women from poor families to get healthcare. If they do not get regular prenatal care to find and monitor any problems during pregnancy, they may have an increased risk of emergency cesarean delivery.

Another factor associated with the risk of CD was the number of children ever born, with CD on first delivery (primary CD) conferring a higher likelihood of future CD. In accordance with other studies, primary CD is the driving force for overall high levels of CD, as primary CD carries an innate risk of repeat CD in future pregnancies [40, 41]. Additionally, multiple pregnancies were identified as a risk factor for CD, which was consistent with other studies [42]. Twin pregnancies are associated with increased perinatal mortality due to a variety of factors, including prematurity‐related complications and birth complications that may contribute to perinatal loss or morbidity [43]. These risk factors justify the option of planned CD to avoid such complications [44]. Beyond that, female babies were less likely to be delivered through CD, as finding shown by other studies as a result of a tendency toward boys having higher birthweights, resulting in fetal pelvic disproportion, which is an indication for CD.

In the univariate analysis, the type of residence was associated with CD, which is consistent with other studies [15, 29, 45], but this association disappeared in the final model, as did employment status, maternal BMI, perceived distance to healthcare facilities, size at birth, and marital status.

### Limitations

This study sheds light on delivery factors for CD in Rwanda but has limitations. This study used cross‐sectional RDHS data, so the causation between identified predictors and CD delivery cannot be established. Second, the RDHS uses self‐reported data, which may be affected by recall bias or social desirability bias. However, the novel findings on CD determinants in Rwanda can be used alongside future work to help improve maternal and newborn health.

### Conclusion

This study on CD prevalence and associated factors underlying the use of CD provides critical information for obstetric decision‐making for this potentially life‐saving surgical procedure. The WHO recommends a CD prevalence of no more than 10%; however, the rate for Rwanda is 15%. This study found associations between CD and being primiparous, delivering male babies, and giving birth in a private healthcare facility. The high CD rates in our study do not necessarily imply the best maternal–child health outcomes. Obstetric and non‐obstetric indications for CD should be evaluated in health facilities with high CD rates. Guidelines should be provided for CD decision‐making, and their implementation should be monitored. Further hospital‐based and qualitative research is needed to better understand the birthing preferences of women and the factors that influence them.

## AUTHOR CONTRIBUTIONS

*Conceptualization and design*: Etienne Nsereko, Marie Josee Mwiseneza, and Patricia Moreland. *Data analysis*: Etienne Nsereko, Aline Uwase, Henriette Usanzineza, and Jean Pierre Niyitegeka. *Drafting the manuscript*: Ornella Mpinganzima, Jean Léonard Turabayo, and Girimpundu Candide Mugani. *Editing manuscript and critical revisions*: Etienne Nsereko and Patricia Moreland. All authors approved the final version submitted.

## CONFLICT OF INTEREST STATEMENT

There are no conflicts of interest to declare.

## FUNDING INFORMATION

There was no funding for the analysis of this dataset.

## ETHICS STATEMENT

The RDHS was approved by the institutional review board, and access to the daset was granted via an online application.There was no attempt to track participants.

## Data Availability

The dataset used to generate the results for this research is available online at the DHS database at https://dhsprogram.com/data/Using‐DataSets‐for‐Analysis.cfm.
